# Evaluation of Remineralization Potential of Beverages modified with Casein Phosphopeptide-Amorphous Calcium Phosphate on Primary and Permanent Enamel: A Laser Profiler Study

**DOI:** 10.5005/jp-journals-10005-1475

**Published:** 2017-02-01

**Authors:** Nitya Rai, Meera Sandhu, Vinod Sachdev, Rina Sharma

**Affiliations:** 1 Postgraduate Student, Department of Pediatric Dentistry, I.T.S Dental College Ghaziabad, Uttar Pradesh, India; 2 Professor, Department of Pediatric and Preventive Dentistry, Maharishi Markandeshwar College of Dental Sciences & Research Maharishi Markandeshwar University, Ambala, Haryana, India; 3 Professor and Head, Department of Pediatric Dentistry, I.T.S Dental College Ghaziabad, Uttar Pradesh, India; 4 Associate Professor, Department of Nanoscale Measurement, National Physical Laboratory, New Delhi, India

**Keywords:** Carbonated beverages, Casein phosphopeptide-amorphous calcium phosphate, Erosion, Remineralization.

## Abstract

**Purpose:**

To assess the erosive potential of carbonated beverages and the remineralization potential of beverages with added casein phosphopeptide-amorphous calcium phosphate (CPP-ACP) paste on primary and permanent enamel.

**Materials and methods:**

A total of 32 primary and 32 permanent enamel specimens were immersed for 5, 10, and 30 minutes, respectively, in beverages, i.e., Coke, Sprite, Mirinda, and Mountain Dew, with and without added 0.2% CPP-ACP. Samples were profiled before immersion and after immersion under laser profiler.

**Results:**

Coke was found to be highly erosive at it caused significant enamel erosion at both 10 and 30 minutes of immersion (p < 0.05) for both primary and permanent enamel. The 30-minute immersion caused significant amount of reminerali-zation over primary enamel in all groups, whereas permanent enamel remineralization was significant in Sprite and Mountain Dew at 30 minutes in all the groups (p < 0.05).

**Conclusion:**

This study demonstrated that enamel erosion occurred after immersion in carbonated beverages. Remineralization of enamel was observed after immersion in beverages modified with CPP-ACP paste. Primary enamel was susceptible to remineralization compared with permanent enamel. Within the limitations of this in vitro study, the application of CPP-ACP paste may enhance the remineralization after an erosive challenge and thus offer some protection for patients who are at risk for erosion.

**How to cite this article:** Rai N, Sandhu M, Sachdev V, Sharma R. Evaluation of Remineralization Potential of Beverages modified with Casein Phosphopeptide-Amorphous Calcium Phosphate on Primary and Permanent Enamel: A Laser Profiler Study. Int J Clin Pediatr Dent 2018;11(1):7-12.

## INTRODUCTION

Dental erosion is the irreversible loss of tooth structure due to chemical dissolution by acids which is not of bacterial origin. It has emerged as the most common chronic disease of children aged between 5 and 17 years, although it is only relatively recently recognized as a dental health problem. In a 5-year-old child, the reported prevalence was found to be 28.57%,^1^ while in an 8 to 19-year-old, it was 30.4%.^2^

The most important sources of tooth-eroding acids are those found in the diet, such as acidic foods and drinks.^3^ Clinical studies have found carbonated drinks to be associated with enamel erosion due to their low pH.^4^ In general, foods and drinks with a pH below 5.0 to 5.7 have been known to initiate dental erosion effects. Acidic foods and drinks have a pH value ranging from 2.5 to 3.4, and consumption of these may lead to tooth demineral-ization. However, susceptibility to dental erosion varies among individuals due to factors, such as pH, salivary flow and buffering capacity, and pellicle formation. The underlying acidity is believed to be the major factor in the development of dental erosion, and the titratable acidity (TA), rather than the pH, is considered to be an important factor in erosion, as it determines the actual hydrogen ion availability for interaction with tooth surface.

Enamel constitutes inorganic calcium and phosphate in the form of hydroxyapatite crystals. During erosion of enamel, fluorhydroxyapatite dissolves, leading to the dissolution of the enamel layer.^5^ However, remineraliza-tion of enamel lesions requires the presence of partially demineralized apatite crystals that grow to their original size when exposed to supersaturated solutions for significant periods of time.^6^

Casein phosphopeptide-amorphous calcium phosphate nanocomplexes have been shown to be readily soluble in saliva, creating a diffusion gradient that allows them to enter the lesion fluid as an intact complex or by releasing the ions in the plaque fluid to then diffuse into the lesion. It is thought that the CPP binding to the apatite crystal faces in the surface of the lesion keeps the diffusion pathways open to allow ions to penetrate more deeply, which results in remineraliza-tion throughout the body of the lesion rather than just in the surface layer.^7^

We hypothesized that incorporation of CPP-ACP by 0.2% w/v in carbonated beverages may reduce enamel erosion, and result in remineralization of primary and permanent enamel. Hence, this study was undertaken to evaluate the effect of CPP-ACP added to carbonated beverages on enamel remineralization of primary and permanent teeth using a noncontact optical profiler.

## MATERIALS AND METHODS

The study design and protocol was approved by the ethical committee of the institutional review board. A total of 64 extracted human teeth with relatively planar buccal and/or lingual surfaces, free from cracks or other artifacts, were included in the study divided into two groups, group I: primary (n = 32), group II: permanent (n = 32). Roots were sectioned at the cementoenamel junction using a carborundum disk and a high-speed water-cooled hand piece. Teeth were then autoclaved for 40 min. Prophylaxis was done using prophylactic paste. Crowns were then sectioned into buccal and lingual halves, using carborundum disk and a straight hand piece under water irrigation. Samples were then embedded in wax blocks. Windows measuring 3 mm^2^ were created by painting the enamel surface with acid-resistant nail varnish.

### Preparation of Test and Control Solutions from Beverages

Four different commercially available carbonated beverages were included in the study, divided into four groups: group I: Coke, group II: Mirinda, group III: Sprite, and group IV: Mountain Dew. Eight enamel specimens from both primary and permanent groups were assigned randomly to each group of beverage. Four enamel specimens of each were then immersed in the two solutions, i.e., without CPP-ACP and with 0.2% CPP-ACP.

The CPP-ACP was supplied by RECALDENT™ (GC India Dental). Beverage with added CPP-ACP paste at 0.2% (w/v) was prepared by dissolving 1 mg of CPP-ACP paste in 5 mL of solution. The beverages were then poured in coded containers so that the researchers were blinded to their identity. Four enamel specimens were immersed in each of 5 mL of solution of respective beverages, with and without added CPP-ACP paste, for 5, 10, and 30 minutes respectively.

**Figs 1A and B:**
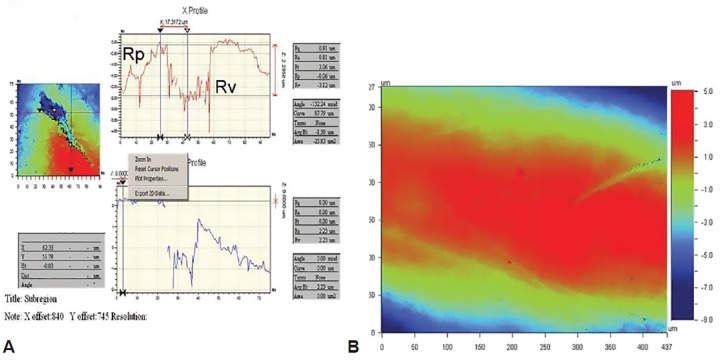
(A) Graphical presentation of surface peak (Rp) and valley (Rv). (B) Enamel surface data with color representation

### Profilometry

The enamel window was profiled before immersion and after each 5, 10, and 30 minutes of immersion, under Wyko 9800 noncontact optical profiler. As the specimens were profiled, the representative depths R_p_, R_v_, and R_z_ were recorded from the profiles generated by the associated software.

### Surface Measurement

To calculate the surface height, the mean height of total peaks and valleys, i.e., R_p_ and R_v_ (Fig. 1), was included. The peak height is the highest point of the peak from the baseline present on the surface of enamel, and the mean of all peak is calculated as R_p_. The valley depth is the deepest point on the surface of enamel from the baseline, and the mean of all valley depths is calculated as R_v_. The mean of R_p_ and R_v_ was then calculated as R_z_. The red area in the software represents the highest enamel surface following green and the lowest area as blue region (Fig. 1).

### pH and Titratable Acidity

The pH value of the solution was measured using a pH meter (Simtronics Analytical & Laboratory Instrument).

The TA of the solutions was measured by dissolving 5.6 gm of KOH in 1 L of distilled water and titrating it against 10 mL of the test solution, until the solution turned pink.

## RESULTS

The present study evaluated the erosion and reminerali-zation potential of beverages with and without added CPP-ACP. A profilometric analysis was used to determine the lesion depth of enamel before immersion and after immersion in each group.

Statistical analysis was done using one-way analysis of variance (ANOVA), Tukey’s honest significant difference (HSD) test, and Student’s t-test, using Statistical Package for the Social Sciences version 16.

The paired t-test was done to statistically analyze the mean erosion depth before and after immersion in each group. The value of significance was set at p < 0.05. On applying the t-test, significant erosion was seen at the 10-minute immersion in Coke for both primary and permanent enamel (p < 0.05). The 30-minute immersion resulted in significant enamel erosion in all beverages on the primary enamel, whereas, on the permanent enamel, significant enamel erosion was seen in Coke and Mirinda over the 30-minute immersion (p < 0.05).

On applying the paired t-test, remineralization over primary enamel was significant in all groups at 10- and 30-minute interval, except at the 10-minute immersion in Mirinda. On permanent enamel, remineralization was found to be significant in Sprite and Mountain Dew at both 10- and 30-minute intervals, whereas in Mirinda, remineralization was significant only at the 10-minute immersion (Table 1).

On applying one-way ANOVA, significant erosion and remineralization were observed on both primary and permanent enamel (Table 2). Analysis of variance followed by Tukey’s *post hoc* test was done. On applying Tukey’s HSD test, a significant difference in the erosion was observed between Coke and Mirinda, and also when Coke and Mirinda were compared with Sprite and Mountain Dew for the primary teeth. On permanent enamel, the difference was significant when Coke was compared with all other beverages. Remineralization was significant for primary teeth, when Coke was compared with all other beverages. On the permanent enamel, remineralization was significant when Coke was compared with Sprite and Mirinda.

A 5-minute immersion did not initiate enamel erosion, for both primary and permanent enamel.

### pH and Titratable Acidity

There was a significant increase in pH of test solutions and a significant reduction in TA, with the addition of CPP-ACP for all beverages included (Table 3).

**Table 1:** **Table 1:** Erosion and remineralization following immersion in beverages with and without added CPP-ACP

				*Erosion (mean ± standard deviation)*				*Remineralization (mean ± standard deviation)*			
*Time (min)*		*Beverage*		*Primary*		*Permanent*		*Primary*		*Permanent*	
5-10		Coke		0.615 ± 0.01*		1.18 ± 0.67*		0.255 ± 0.10*		0.305 ± 0.27 NS	
10-30		Coke		0.275 ± 0.05*		0.582 ± 0.22*		1.07 ± 0.26*		0.475 ± 0.36 NS	
5-10		Mirinda		0.542 ± 0.36 NS		0.422 ± 0.47 NS		0.942 ± 1.05 NS		0.585 ± 0.11*	
10-30		Mirinda		0.255 ± 0.10*		0.17 ± 0.10*		1.05 ± 0.66*		1.90 ± 1.35 NS	
5-10		Sprite		-		-		0.135 ± 0.04*		0.742 ± 0.24*	
10-30		Sprite		0.615 ± 0.32*		0.087 ± 0.36 NS		0.45 ± 0.16*		1.18 ± 0.67*	
5-10		Mountain Dew		-		-		0.06 ± 0.03*		0.18 ± 0.08*	
10-30		Mountain Dew		0.305 ± 0.09*		0.542 ± 0.36 NS		0.277 ± 0.05*		0.255 ± 0.04*	

*p < 0.05; NS: Nonsignificant, - no erosion

**Table 2:** **Table 2:** Comparison of primary and permanent teeth with and without added CPP-ACP for 30 minutes

		*Erosion (mean ± standard deviation)*				*Remineralization (mean ± standard deviation)*			
*Beverage*		*Primary*		*Permanent*		*Primary*		*Permanent*	
Coke		0.38 ± 0.24		0.59 ± 0.50		0.78 ± 0.58		0.78 ± 0.20	
Mirinda		0.92 ± 0.18		18 ± 0.72		2.24 ± 1.5		2.49 ± 1.4	
Sprite		0.67 ± 0.42		0.09 ± 0.36		0.58 ± 0.13		1.87 ± 1	
Mountain Dew		0.25 ± 0.08		0.30 ± 0.05		0.34 ± 0.04		0.43 ± 0.06	
p-value		0		0		0		0.002	

**Table 3:** **Table 3:** Change in pH and TA of beverages following addition of CPP-ACP

*Beverage*		*pH without added 0.2% CPP-ACP*		*pH with added 0.2% CPP-ACP*		*Mean*		*TA without added 0.2% CPP-ACP*		*TA with added 0.2% w/v CPP-ACP*		*Mean*	
Coke		2.81		3.11		0.302 ± 0.03*		0.125		0.085		0.04 ± 09*	
Mirinda		2.95		3.15		0.202 ± 0.03*		0.190		0.155		0.04 ± 0.02*	
Sprite		3.57		3.82		0.247 ± 0.04*		0.147		0.105		0.04 ± 0.01*	
Mountain Dew		3.24		3.46		0.217 ± 0.04*		0.165		0.115		0.05 ± 0.08*	

## DISCUSSION

Enamel is the outermost hard tissue of the tooth crown and is known to be the most highly mineralized tissue in the human body. Wang^8^ investigated the enamel demin-eralization in primary and permanent teeth. During dissolution, crystallites became smaller, and eventually nanosized crystallites attached to the primary enamel surface or escaped into the bulk solution. After further dissolution, enamel walls of the primary teeth tended to fracture, while permanent enamel walls remained intact.

**Figs 2A and B:**
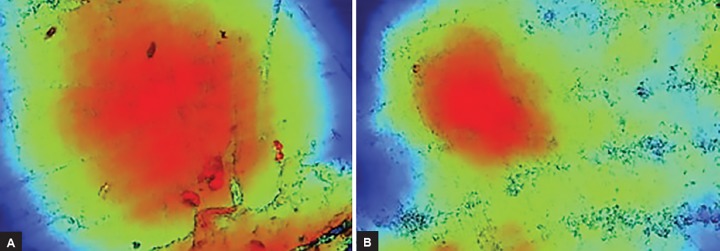
Erosion following immersion in beverages without added CPP-ACP. (A) Preimmersion; (B) postimmersion

The present study investigated the erosion and rem-ineralization potential of primary and permanent enamel immersed in carbonated beverages with and without added 0.2% w/v CPP-ACP paste. Erosion was observed following immersion in beverages without added CPP-ACP (Fig. 2). For the primary enamel, a 10-minute exposure caused significant erosion of enamel, in Coke and Mirinda (p < 0.05), and an increase in erosion by 30 minutes in all the groups (p < 0.05), i.e., represented by a decrease in the R_z_ value as erosion caused the decrease in the mean surface height after immersion (Tables 1 and 2). This could be attributed to the reason that the thickness of the enamel layer does not exceed 1 mm in primary teeth and is susceptible to acidic attack easily. Deciduous teeth demonstrate a higher degree of enamel porosity^9^ and a lower degree of mineralization^10^ than permanent teeth. This was attributed to the greater density of the interprismatic fraction and the prism-junction in deciduous enamel than its permanent analog.^11^ In the primary dentition, it is thought that the reduced thickness of enamel and greater acid solubility contribute to the higher susceptibility to erosion.

A high degree of mineralization is present in the permanent enamel compared with the primary enamel.^10^ According to the results of this study, a 10-minute immersion in Sprite and Mountain Dew resulted in the erosion on the primary enamel, whereas no reduction in the enamel depth was observed in the permanent enamel for the 10-minute immersion in Sprite and Mountain Dew.

The erosive loss of dental hard tissue associated with excessive consumption of carbonated beverages occurs due to various acids. Citric and phosphoric acids are the two main dietary acids present in these soft drinks. One of the basic ingredients of Coca-Cola is phosphoric acid, and citric acid is also added for flavor modification.^12^ Whereas in Mirinda, citric acid is known to initiate demin-eralization of enamel, as citric, malic, and tartaric acids are considered to be especially erosive because of their acidic nature and the ability to chelate calcium at higher pH.^13^ West et al^14^ investigated the effect of pH on the erosion of dentin and enamel by dietary acids *in vitro* and found that phosphoric acid caused far more erosion over a period of 30 minutes than citric acid for both enamel and dentin. Similarly, in this study, Coca-Cola initiated erosion at the 10-minute immersion. Though Mirinda contains citric acid that caused pronounced loss at 10 minutes (p < 0.05), there is no significant difference in enamel loss at the 30-minute immersion (p > 0.05). Hence, this justifies that citric acid, also being a strong acid, is capable of initiating erosion early, and Sprite and Mountain Dew, in spite of containing citric acid, did not initiate surface erosion at the 10-minute interval; this could be attributed to the less amount of citric acid incorporated in these beverages.

**Figs 3A and B:**
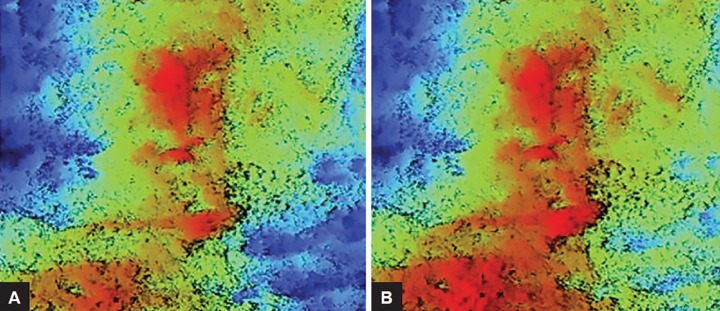
Remineralization following immersion in beverages with added CPP-ACP (A) Preimmersion; (B) postimmersion

Significant remineralization was observed over primary enamel in all the tested beverages with added CPP-ACP, whereas on permanent enamel, significant remineralization was seen after immersion in Sprite and Mountain Dew (Tables 1 and 2). As CPP-ACP was incorporated in beverages at 0.2% w/v, an increase in the mean surface height, i.e., R_z_ was observed following remineralization of enamel (Fig. 3). This further explains that there was precipitation of calcium and phosphate over peak as the peak height increased and no remineralization in the deep valleys of enamel surface; hence, it protects the enamel from further dissolution due to acid attack. The CPPs stabilize calcium and phosphate, keeping them in the amorphous form, i.e., biologically active, known as ACP. The CPP-ACP increases the remineralization of the eroded enamel.^15,16^ According to these results, CPP-ACP-containing products can be a substitute for preventing dental erosion.

Poggio et al^17^ conducted a study to measure the surface roughness using atomic force microscopy. According to the results, the superficial layer of the rem-ineralizing agent was probably constituted by a globular arrangement of the mineral substances. Similarly, in our study, it was observed that remineralization of calcium and phosphate precipitates occurred over the superficial layer of enamel, i.e., increase in the R_z_ value.

NT9800 Optical Profiler, which is the most advanced optical interferometric profiler, was used in the study. The Wyko NT9800 Optical Profiler is used for rapid measurement of step heights, surface roughness, and a host of other topographical characteristics. The measurement of steps, i.e., valley, and heights, i.e., peak, were included in the study, as erosion can cause reduction in height by removing the superficial surface, whereas remineralization may result in deposition of precipitates in the steps or above the heights. As seen in our study, remineralization occurred only over the heights and not in the steps. Thus, it is concluded that remineralization occurs only over the superficial surface of enamel and not in the deeper enamel layer. Therefore, it protects the enamel by forming a protective layer from further dissolution.

The pH is the negative logarithm of the hydrogen ion concentration (actual hydrogen ion concentration). Beverages with lower pH values i.e., less than 7, have greater erosive effect. The TA level may be a more accurate method for measuring the potential acidity in a given beverage, as it is the total number of acid molecules, and the actual hydrogen ion available for interaction with the tooth surface.^18^

**Graph 1:**
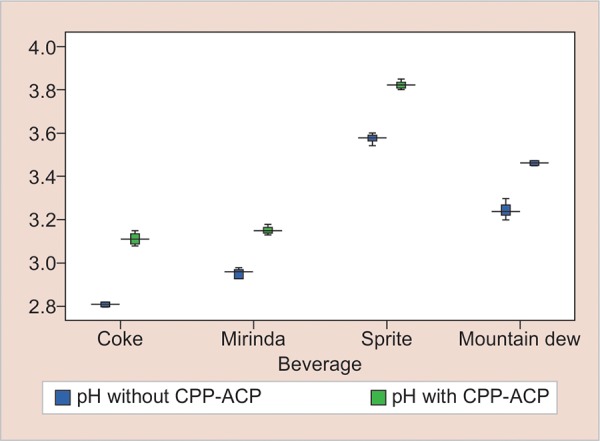
Distribution of mean change in pH of beverages following incorporation of CPP-ACP

In the present study, the pH of the solutions increased by an increment of approximately 0.3 units (Graph 1), and TA decreased by an increment of 0.4 units, after the addition of 0.2% CPP-ACP (Table 3). However, all beverages remained below pH 3.57, which would still be expected to erode tooth enamel *in vivo.* Even at the low pH values, the test solutions with 0.2% CPP-ACP were not erosive and caused remineralization over the enamel surface.

Other factors involved with the erosion process include the type, concentration, amount of acid, calcium chelating properties, exposure time and temperature, and the buffering capacity of saliva or flow rate and/or saliva content. The exposure time also depends on the method of drinking, as holding the drink in the mouth before swallowing leads to the most pronounced pH drop followed by the long-sipping method.^19^ As the exposure time is an important variable in the extent of erosion, and in young adolescence, individuals are more prone to longer exposure due to the method of drinking, i.e., long holding and squishing before swallowing. Therefore, the exposure time of 10 to 30 minutes was included in this study.

The present study utilized an *in vitro* model, and therefore, the findings cannot be hypothesized to the *in vivo* state, as intraoral constituents, i.e., saliva and pellicle, were excluded. Therefore, as the addition of CPP-ACP to the drinks decreased the erosive potential in this *in vitro* model, it is likely to be more effective *in vivo.* The addition of CPP-ACP to carbonated beverages may thus help in reducing the erosive potential of acidic beverages.

## CONCLUSION

- Immersion in beverages without CPP-ACP resulted in erosion for both primary and permanent enamel. Incorporation of 0.2% CPP-ACP resulted in the rem-ineralization of primary and permanent teeth.
- Coke was found to be highly erosive for both primary and permanent enamel.
- Erosion was found to be time-dependent as the lesion depth increased with immersion time.
- The primary enamel showed a higher remineralization potential compared with permanent enamel.
- The laser profiler was found to be an efficient way to qualitatively assess the enamel surface characteristics during an *in vitro* study.

## CLINICAL SIGNIFICANCE

This study helps to assess the erosive potential of various carbonated beverages included in the study, whereas prevention of erosion by incorporation of CPP-ACP helps in stabilizing calcium and phosphate in an amorphous form, which enters the erosive lesion as an intact complex, and helps in remineralization.
